# Pharmacological smoking cessation of adults aged 30–50 years with COPD

**DOI:** 10.1038/s41533-022-00301-y

**Published:** 2022-10-08

**Authors:** Dea Kejlberg Andelius, Ole Hilberg, Rikke Ibsen, Anders Løkke

**Affiliations:** 1 Research Unit for General Practice, Aarhus, Denmark; 2 Department of Medicine, Little Belt Hospital, Vejle, Denmark; 3 Department of Regional Health Research, University of Southern Denmark, Odense, Denmark; 4 i2minds, Aaboulevarden 39, 8000 Aarhus, Denmark

**Keywords:** Epidemiology, Disease prevention

## Abstract

The prevalence of active smokers has remained relatively stable around 20% for several years in Denmark despite knowledge of the harmful effects. Smoking cessation is the most effective way to limit progression and reduce mortality of chronic obstructive pulmonary disease (COPD). Therefore, smoking cessation is particularly important among adults with COPD. The aim of this study was to determine the extent to which adults 30–50 years of age with COPD redeem pharmacotherapy for smoking cessation, and to identify demographic factors that influence the use of smoking cessation medication. We conducted a national retrospective non-interventional registry study, including all Danish patients with COPD (ICD-10 code J.44: chronic obstructive pulmonary disease) aged 30–50 years in the period 2009–2015. We identified 7734 cases, who were matched with controls (15,307) 1:2 on age, sex, and geography. Smoking status was not registered. We found that 18% of cases (with an estimated smoking prevalence at 33–50%) redeemed pharmacological smoking cessation medication in the study period compared to 3% of the controls (with an estimated smoking prevalence at 23%). The OR for cases collecting pharmacological smoking cessation medication was 5.92 [95% CI 5.24–6.70]. Male sex, being unemployed, and receiving social benefits were factors associated with less probability of redeeming pharmacological smoking cessation medication. Our study indicates that attention is needed on smoking cessation in adults aged 30–50 years with COPD, especially if unemployed or receiving social benefits, as these individuals are less likely to redeem pharmacological smoking cessation medication.

## Introduction

Tobacco smoking is the leading cause of preventable death in the western world^1^. The overall mortality for people who smoke is three times higher than for people who have never smoked^2^. According to the Danish Health Authorities, tobacco smoking is the most influential factor for inequalities in health and mortality in Denmark^3^. Smoking is known to cause multiple diseases, e.g., lung cancer, cardiovascular disease, and chronic obstructive pulmonary disease (COPD)^4,5^. Without smoking cessation, the lifetime risk of developing COPD is one out of two^6^.

Due to extensive cigarette use, the prevalence and mortality of COPD in Denmark are among the highest in Europe^7^. Danish women have a 3.7 times higher risk of dying from COPD than the general European population^7^. Therefore, there is a great need to improve treatment and reduce the mortality of Danish patients with COPD.

COPD can be treated through pharmacological and non-pharmacological interventions^8,9^. The most effective way to inhibit disease progression is smoking cessation^10–12^. Smoking cessation in patients with COPD is associated with decreased dyspnea, fewer exacerbations and hospitalizations, better lung function, increased quality of life, and increased survival^13,14^. Despite the positive effects of smoking cessation, ~33% of Danish patients with moderate or severe COPD are smoking^15,16^. Smoking cessation is essential in young adults since their lungs are more vulnerable to the harmful effects of smoking^17^. Hospitalization and mortality rates are increased in patients diagnosed with COPD at an early age (before 50 years of age) compared to patients who are diagnosed with COPD after 50 years of age^18^. Even though smoking cessation is the most effective way to inhibit disease progression, a recent study found that up to 50% of Danish patients with COPD under 50 years of age are active smokers^18^. One reason for the extensive tobacco use among young patients could be that they are more nicotine dependent and thus face more difficulties with smoking cessation than older patients with COPD^16^.

Three main strategies exist for smoking cessation: quitting without external aid, quitting by the help of professional counseling (e.g., motivational therapy), or quitting by means of smoking cessation medication^19^. In recent years smoking cessation by means of electronic cigarettes has been promoted, but the products have not been approved as pharmacological treatment, and the effect on smoking cessation rates is not well established^20^. Pharmacological treatment has proved to be more efficient than the other strategies, although successful smoking cessation is best achieved through a combination of counseling and pharmacological treatment^19,21^. Three main types of smoking cessation medication are available: nicotine replacement therapy (NRT), bupropion, and varenicline. Quitting rates varies between studies, but are ~20–30% for varenicline, 15–40% for NRT, and 10–20% for bupropion^22–26^.

COPD is a disease with a heavy socioeconomic gradient and socioeconomic status is considered an independent risk factor for the development of COPD^27^. The cost of the recommended 12-week treatment varies but is approximately EUR 120 for bupropion (Zyban^®^, GlaxoSmithKline Pharma) and EUR 360 for varenicline (Champix^®^, Pfizer). The price of NRT varies, depending on the dosage and duration of the treatment. In Denmark, as in several other European countries, smoking cessation medication is not covered by the national reimbursement system, and other means of financial support is limited^28^. In some municipalities in Denmark (including our study period 2009–2015), smoking cessation medication has experimentally been offered, for various time periods, free of charge to specific groups, e.g., heavy smokers, economically challenged individuals, and pregnant women, if they participated in smoking cessation programs offered by the Danish municipalities^29^. These programs have been a success but smoking cessation medication is still not part of the national reimbursement system^30^.

It is of great importance to increase focus on smoking cessation among young adults. A study from 2019 by Tibuakuu et al. showed that tobacco-dependent adults (18–39 years of age) in the United States received less smoking cessation advice from physicians than the rest of the population^31^. Although pharmacological smoking cessation has been proven effective there is still limited knowledge on the degree to which smoking cessation medication is offered to patients with COPD, particularly in young patients^32,33^.

The aim of this study was to determine the extent to which adults aged 30–50 years with COPD redeem pharmacotherapy for smoking cessation, and to explore how the use of smoking cessation medication is correlated to different demographic factors.

## Methods

### Study design

The study was designed as a national, retrospective, non-interventional, registry study.

### Data collection

The Danish Civil Registration System (CPR) is a national register that contains information on all Danish citizens. All residents in Denmark receive a unique 10-digit (CPR) number which is recorded in the Danish Civil Registration System. The CPR number enabled us to link data at an individual level across different registries^34^.

We included all Danish patients 30–50 years of age who were diagnosed with COPD in the period 2009–2015. Data were collected from the Danish National Patient Registry, which is a complete nationwide registry covering all non-psychiatric contacts to the secondary healthcare sector in Denmark^34^. We used code J44.x of the International Classification of Diseases 10^th^ revision (ICD-10) to identify patients with COPD. Cases and controls were included regardless of smoking status, since this information was not available from any national registry. We excluded patients who died in 2009 to allow for at least one year of eligibility to collect pharmacological smoking cessation medication.

Each case was matched with two controls without a COPD diagnosis in the period 1998–2015. We matched cases and controls on age, sex, and geography. We used descriptive statistics from the index year (2009).

We extracted data on age, sex, and geography from the Danish Civil Registration System^35^. Information about socioeconomic status, educational level, and marital status was obtained from Statistics Denmark. All redeemed prescriptions are registered in the Danish National Prescription Registry and can be linked to the individual CPR number^36^. We included all redeemed prescriptions with codes N07BA01 (NRT), N06AX12 (bupropion), and N07BA03 (varenicline) of the Anatomical Therapeutic Chemical (ATC) Classification System^37^. NRT can be bought as over-the-counter medicine in Denmark. All medication sold as over-the counter medicine is not recorded in the national registries and therefore not included in the study. This applies only for NRT and not for varenicline or bupropion.

**Fig. 1:**
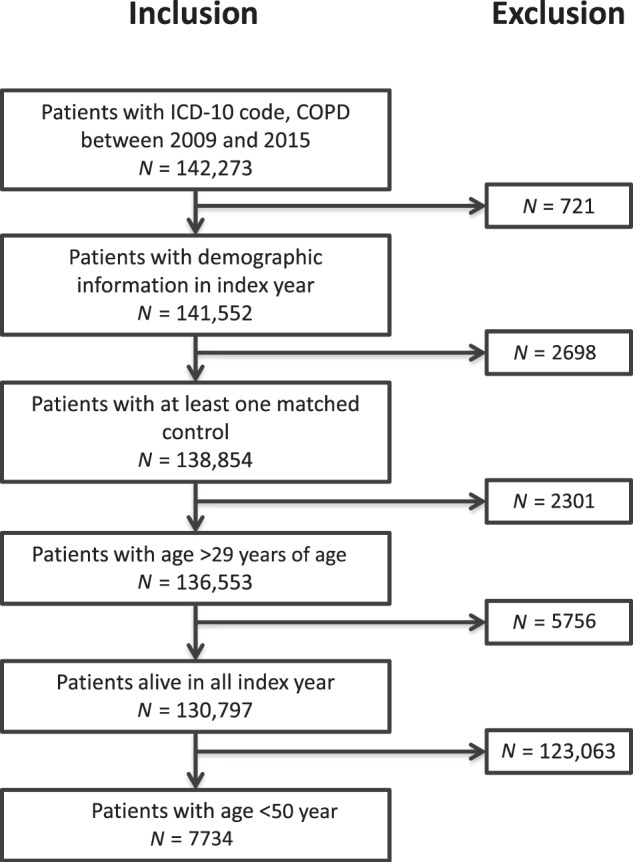
Inclusion process.

Figure 1 shows the inclusion process. A total of 142,273 cases with a COPD diagnosis in 2009–2015 were identified. A total of 721 were excluded due to missing demographic information, and 2698 cases were excluded because we were unable to identify at least one control. An additional 5756 cases were excluded because they died in the index year, and 123,063 cases were excluded due to age above 50 years. The remaining 7734 cases were individually matched with two controls. In total, we included 15,307 controls because we were able to identify only one control in 161 cases.

### Statistics

We used a conditional logistic regression model, with case = 1, control = 0, to compare socioeconomic factors for cases and controls. In the model we corrected for socioeconomic factors by using a variable (SOCIO13) from Statistic Denmark for the labor market affiliation status. The variable is based on information about the most important source of income or employment for the person in the year^38^. We used SAS 9.4 TS1M5 (SAS, Inc., Cary, NC, USA) to perform the statistical analyses.

A smoking cessation attempt was defined as the redemption of a prescription for smoking cessation medication. If the period between the redemption of two prescriptions on smoking cessation medication exceeded six month plus the number of treatment days of the previous prescription (measured as defined daily dosage (DDD)) it was categorized as a new smoking cessation attempt.

### Outcome

The outcome was the number of redeemed prescriptions for smoking cessation medicine with ATC code N07BA01 (NRT), N06AX12 (bupropion), or N07BA03 (varenicline).

### Ethics

The study was approved by the Danish Data Protection Agency. The design was a register-based study, which was based on anonymized data with no identifiable personal information. Therefore, approval was not required by the Danish health research ethics committee system and consent from patients not necessary according to Danish Law. The study complied with the ethical principles outlined by the World Medical Association in the Declaration of Helsinki.

### Reporting summary

Further information on research design is available in the Nature Research Reporting Summary linked to this article.

## Results

### Demographic characteristics

The demographic characteristics of the cohort are displayed in Table 1. The mean age was 44 years, with an even distribution of males and females. A total of 48.0% of the cases were married compared to 70.7% of the controls. Cases had lower employment rates than controls (42.9% of cases vs. 82.1% of controls). Cases more often received social benefits than controls; 30.4% on disability pension (vs. 6.6% of controls), 4.2% on sick pay (vs. 1.6% of controls), and 17.8% on social security (vs. 4.4% of controls). Cases had lower education level compared to controls; 9.2% of cases had a bachelor’s or higher degree (vs. 26.6% of controls).

**Table 1:** Basic characteristics.

Number of people	Case		Control	
	7734		15,307	
Age	Mean	Std.	Mean	Std.
	44	5	44	5
	*N*	%-share	*N*	%-share
Gender				
Male	3777	48.8	7460	48.7
Female	3957	51.2	7847	51.3
Civil status				
Married/co-living	3711	48.0	10,823	70.7
Single	4023	52.0	4484	29.3
Socioeconomy				
Disability pension	2350	30.4	1011	6.6
Educational support	175	2.3	449	2.9
Employed	3315	42.9	12,570	82.1
Sickpay/leave	321	4.2	244	1.6
Social security	1374	17.8	675	4.4
Unemployment benefit	199	2.6	358	2.3
Education				
Primary	3610	46.7	3064	20.0
Secondary	278	3.6	926	6.0
Vocational	2642	34.2	6113	39.9
Short college	191	2.5	862	5.6
Bachelor	548	7.1	2681	17.5
Master/PhD	161	2.1	1398	9.1
Unknown	304	3.9	263	1,7

### Prescription redemption

Table 2 displays the number of cases and controls that redeemed a prescription for smoking cessation medication. A total of 18.3% of cases redeemed a prescription for smoking cessation medication, whereas only 3.3% of the controls did the same. Among both cases and controls, the age group 40–50 years redeemed more medication than the age group 30–39 years, independently of the subtype of medication. Among cases, 20.9% of females redeemed a prescription for smoking cessation medication, whereas this was 16.2% for males. There were no differences regarding sex in the control group (3.3%).

**Table 2:** Number (n) of cases and controls who redeemed at least one prescription for smoking cessation medication.

	All types			Nicotine Replacement Therapy (NRT)			Varenicline			Bupropion		
	Case	Control		Case	Control		Case	Control		Case	Control	
	*n* (*n/N*, %)	*n* (*n/N*, %)	*P*-value	*n* (*n/N*, %)	*n* (*n/N*, %)	*P*-value	*n* (*n/N*, %)	*n* (*n/N*, %)	*P*-value	*n* (*n/N*, %)	*n* (*n/N*, %)	*P*-value
Number of persons	1441 (18.3)	504 (3.3)		454 (5.9)	61 (0.4)		906 (11.7)	371 (2.4)		287 (3.7)	102 (0.7)	
Age												
30–39 years	215 (14.3)	78 (2.6)	<0.001	68 (4.5)	10 (0.3)	<0.001	125 (8.3)	59 (2.0)	<0.001	48 (3.2)	14 (0.5)	<0.001
40–50 years	1226 (19.7)	426 (3.4)	<0.001	386 (6.2)	51 (0.4)	<0.001	781 (12.5)	312 (2.5)	<0.001	239 (3.8)	88 (0.7)	<0.001
Sex												
Male	613 (16.2)	245 (3.3)	<0.001	206 (5.5)	32 (0.4)	<0.001	374 (9.9)	173 (2.3)	<0.001	116 (3.1)	52 (0.7)	<0.001
Female	828 (20.9)	259 (3.3)	<0.001	248 6.3)	29 (0.4)	<0.001	532 (13.4)	198 (2.5)	<0.001	171 (4.3)	50 (0.6)	<0.001
Civil status												
Married/co-living	667 (18.0)	338 (3.1)	<0.001	133 (3.6)	27 (0.2)	<0.001	483 (13.0)	253 (2.3)	<0.001	129 (3.5)	75 (0.7)	<0.001
Single	774 (19.2)	166 (3.7)	<0.001	321 (8.0)	34 (0.8)	<0.001	423 (10.5)	118 (2.6)	<0.001	158 (3.9)	27 (0.6)	<0.001
Socioeconomy												
Disability pension	547 (23.3)	57 (5.6)	<0.001	305 (13.0)	22 (2.2)	<0.001	254 (10.8)	33 (3.3)	<0.001	86 (3.7)	9 (0.9)	<0.001
Educational support	28 (16.0)	7 (1.6)	<0.001	4 (2.3)	3 (0.7)	0.085	22 (12.6)	4 (0.9)	<0.001	5 (2.9)	0 (0.0)	<0.001
Employed	588 (17.7)	399 (3.2)	<0.001	62 (1.9)	31 (0.2)	<0.001	459 (13.8)	306 (2.4)	<0.001	140 (4.2)	84 (0.7)	<0.001
Sickpay/leave	62 (19,3)	8 (3.3)	<0.001	20 (6.2)	0 (0)	<0.001	39 (12.1)	6 (2.5)	<0.001	11 (3.4)	–	–
Social security	193 (14.0)	21 (3.1)	<0.001	56 (4.1)	3 (0.4)	<0.001	114 (8.3)	14 (2.1)	<0.001	42 (3.1)	5 (0.7)	0.001
Unemployment benefit	23 (11.6)	12 (3.4)	<0.001	7 (3.5)	0 (0)	0.002	18 (9.0)	8 (2.2)	<0.001	3 (1.5)	3 (0.8)	0.463
Education												
Primary	729 (20.2)	146 (4.8)	<0.001	286 (7.9)	24 (0.8)	<0.001	419 (11.6)	106 (3.5)	<0.001	137 (3.8)	26 (0.8)	<0.001
Secondary	37 (13.3)	22 (2.4)	<0.001	14 (5.0)	4 (0.4)	0.112	21 (7.6)	12 (1.3)	<0.001	6 (2.2)	6 (0.6)	<0.001
Vocational	493 (18.7)	226 (3.7)	<0.001	103 (3.9)	19 (0.3)	<0.001	351 (13.3)	171 (2.8)	<0.001	106 (4.0)	49 (0.8)	<0.001
Short college	31 (16.2)	25 (2.9)	<0.001	8 (4.2)	3 (0.3)	<0.001	23 (12.0)	22 (2.6)	<0.001	7 (3.7)	0 (0.0)	<0.001
Bachelor	87 (15.9)	59 (2.2)	<0.001	14 (2.6)	3 (0.1)	<0.001	62 (11.3)	46 (1.7)	<0.001	19 (3.5)	14 (0.5)	0.026
Master/PhD	13 (8.1)	15 (1.1)	<0.001	0 (0.0)	5 (0.4)	<0.001	8 (5.0)	10 (0.7)	0.001	5 (3.1)	0 (0.0)	0.501
Unknown	51 (16.8)	11 (4.2)	<0.001	27 (8.9)	3 (1.1)	<0.001	22 (7.2)	4 (1.5)	<0.001	7 (2.3)	4 (1.5)	<0.001

Low education level was associated with an increased likelihood of redeeming smoking cessation medication among both cases and controls. A total of 20.2% of cases with the primary school as their highest attained education redeemed a prescription, whereas only 8.1% for cases holding a master’s degree redeemed a prescription. A similar pattern was seen for controls, regardless of the type of medication.

Table 3 shows case data only. Male sex reduced the probability of redeeming a prescription for smoking cessation medication (Odds ratio (OR) 0.73 [95% CI (confidence interval) 0.65–0.82]). Civil status did not affect the probability of redeeming a prescription for smoking cessation medication. Cases on social security had the lowest probability of redeeming a prescription for smoking cessation medication (OR 0.75 [95% CI 0.63–0.90]). Employed cases had the highest probability of redeeming a prescription for varenicline or bupropion (OR 1, *reference*).

**Table 3:** Logistic regression for cases redeeming af prescription on smoking cessation medication.

	All types		NRT		Varenicline		Bupropion	
	Odds ratio	*P*-value	Odds ratio	*P*-value	Odds ratio	*P*-value	Odds ratio	*P*-value
Age	1.02 (1.01–1.04)	<0.001	1.00 (0.98–1.02)	0.715	1.04 (1.02–1.05)	<0.001	1.01 (0.99–1.04)	0.391
Sex								
Male	0.73 (0.65–0.82)	<0.001	0.85 (0.69–1.03)	0.096	0.71 (0.61–0.82)	<0.001	0.69 (0.54–0.88)	0.003
Female	–	–	–	–	–	–	–	–
Civil status								
Married/co-habiting	0.90 (0.79–1.02)	0.088	0.62 (0.50–0.77)	<0.001	1.07 (0.92–1.24)	0.388	0.77 (0.60–0.99)	0.038
Single	–	–	–	–	–	–	–	–
Socioeconomy								
Disability pension	1.27 (1.11–1.46)	0.001	6.51 (4.88–8.69)	<0.001	0.70 (0.59–0.83)	<0.001	0.76 (0.57–1.01)	0.059
Educational support	0.91 (0.60–1.38)	0.650	1.22 (0.44–3.41)	0.702	0.94 (0.59–1.50)	0.806	0.65 (0.26–1.62)	0.357
Employed	–	–	–	–	–	–	–	–
Sickpay/leave	1.10 (0.82–1.48)	0.526	3.23 (1.92–5.45)	0.120	0.87 (0.61–1.24)	0.445	0.79 (0.42–1.47)	0.453
Social security	0.75 (0.63–0.90)	0.002	1.97 (1.35–2.86)	<0.001	0.59 (0.48–0.74)	<0.001	0.67 (0.47–0.96)	0.027
Unemployment benefit	0.65 (0.41–1.01)	0.054	1.88 (0.85–4.17)	0.120	0.68 (0.41–1.12)	0.128	0.37 (0.12–1.16)	0.088

### Attempts to quit smoking

Table 4 displays a conditional logistic regression analysis of attempts to quit smoking among cases. The OR for cases to try smoking cessation medication once was 5.59 [95% CI 4.86–6.44] compared to controls, whereas the OR for three attempts was 10.00 [95% CI 2.87–11.06].

**Table 4:** Logistic regression showing attempts to quit smoking.

Attempts to quit	Case (*n*)	Control (*n*)	Odds ratio	*P*-value
0	6293	14,803	–	–
1	1008	377	5.59 (4.86–6.44)	<0.001
2	282	93	6.43 (4.90–8.43)	<0.001
3	104	20	10.00 (2.87–11.06)	<0.001
4	47	14	5.63 (2.87–11.06)	<0.001

## Discussion

In this retrospective non-interventional registry-based study, we provide national data regarding adults 30–50 years of age with COPD. In a previous publication we addressed the pharmacological smoking cessation in patients with COPD of all ages, but in our present study we included only patients with early onset COPD, since there is a big knowledge gap regarding the treatment of this particular group of patients. In accordance with a previous study on all Danish patients with COPD, we found a higher likelihood among patients with COPD to redeem prescriptions for smoking cessation medication than among their matched controls^39^. We found that cases had an inferior social situation compared to controls. Cases were more likely to be single, have low education level, and to be on social benefits. These characteristics have also been found in other studies^32,40–42^. Furthermore, our study showed that among cases, male sex and receiving social security decreased the likelihood to redeem a prescription for smoking cessation medication. The inferior socioeconomic situation of the patients with COPD, lack of smoking cessation advice, and higher nicotine dependence might explain the low redemption rate of smoking cessation medication, but more studies are needed to identify contributing factors^16,32,43^.

We found that 18.3% of cases redeemed a prescription for smoking cessation medication at least once compared to only 3.3% of controls. Even though cases used more smoking cessation medication than controls, the number is discouraging. Young patients (under 50 years of age) with COPD have increased risk of hospitalizations and reduced life expectancy compared to their lung healthy peers^18^. Given the poor prognosis and the prospects of a life with potentially severe pulmonary symptoms, young patients with COPD is expected to be more motivated to smoking cessation. However, patients with early COPD often have fewer respiratory symptoms and are known to have more psychiatric comorbidities, which could be potential reasons for the limited use of smoking cessation medication in this group. Further, in Denmark there are no tailored smoking cessation programs for young individuals.

We found that cases were five times more likely to have one quit attempt (OR 5.59) and 10 times more likely to have three quit attempts (OR 10.00) compared to controls. There may be numerous reasons for this finding. First, COPD patients with symptoms of airway obstruction are more likely to stop smoking^44^. Second, young patients with COPD may have been advised to stop smoking during their annual routine checkup. Advice on smoking cessation is a mandatory part of the annual routine checkup according to the Danish COPD guidelines, and discussing smoking cessation with a health care professional has been shown to increase the chance of successful smoking cessation and prescription of relevant medications^45–47^. Lastly, the excessive attempts to quit smoking among cases as compared to controls may reflect a higher level of nicotine dependence among patients with COPD, resulting in difficulties with smoking cessation^16,48^.

Patients with COPD had the highest probability of redeeming a prescription of either varenicline or bupropion if they were employed. This might be due to the high prices of especially varenicline and bupropion. Surprisingly, we found that cases on early retirement had an OR of 6.51 for redeeming a prescription of NRT as compared to employed cases. A possible explanation for this finding could be that people on early retirement may prioritize medication that is available in small portions, which makes it more affordable in the short term. It may also be because they are reimbursed by some municipalities in Denmark as a part of a smoking cessation reimbursement program. Adding smoking cessation medication in the national reimbursement program, or even offering the medication free of charge, could be cost-effective strategies to help patients with COPD to smoking cessation. Studies from other countries, such as Spain, the United Kingdom, and the Netherlands have showed, that funding of smoking cessation medication may increase quitting rates^49–51^.

The socioeconomic gradient in COPD remains a challenge, which may contribute to a delayed diagnosis, inferior medical treatment, and a poor prognosis^32,43^. Despite the well-known socioeconomic gradient of COPD, we were surprised to find that only 42.9% of adults aged 30–50 years with COPD were employed (compared to 82.1% of controls) and that 30.4% were on disability pension (compared to 6.6% of controls). Similar findings have been reported from other contries^52–54^. It is discouraging that so many adults aged 30–50 years with COPD experience such socioeconomic disadvantages, and it underlines the importance of early diagnosis and targeted treatment to inhibit disease progression.

We found that sex was related to collection of smoking cessation medication. Males redeemed significantly fewer prescriptions for smoking cessation medication compared to females. In Denmark, the prevalence of daily smoking is similar between males and females, so differences in smoking status cannot explain this finding^55^. A study by Watson et al. showed that women were more likely to experience dyspnea and more likely to take smoking cessation advice (OR 1.52)^56^. Therefore, a likely explanation could be that women redeem more prescriptions for smoking cessation medication because they experience more symptoms and seek advice on smoking cessation from health care professionals.

A limitation to this study is that we were not able to collect information about smoking status among cases and controls since this information is not routinely registered in any Danish database. According to the Danish COPD database, 33% of patients with COPD were current smokers in 2015, but other studies suggest, that the smoking prevalence among patients with COPD under the age of 50 years is as high as 50%^18^. The number was 23% for the general population in the same period. If we take the smoking prevalence among cases and controls into account, we find that ~36–55% (18 out of 33–50) of active smokers with COPD redeem a prescription on smoking cessation medication compared to 15% (3,3 out of 23) among smokers without COPD.

A strength of our study is the use of a national dataset on all Danish patients with an ICD-10 code for COPD. Unfortunately, the primary care sector in Denmark does not use ICD-10 coding. This means that patients with no contact to a Danish hospital or outpatient clinic is not included in the study. Still, this number is likely to be very low as the Danish Health Authorities recommend referral of all patients under 50 years of age diagnosed with COPD for further checkup with a pulmonary specialist^57^.

We could not validate the COPD diagnoses of the included cases since we had no access to lung function data. However, a previous study by Thygesen et al. found a positive predictive value of nearly 100% for a COPD diagnosis in the Danish National Patient Registry^58^.

The results on the use of NRT should be interpreted with caution. The majority of NRTs are sold as over-the-counter medicine in Denmark. Therefore, NRT are not recorded in the national registries. However, the use of over-the-counter NRTs should not differ between the case group and the control group.

We found that only 18% of young patients 30–50 years of age diagnosed with COPD redeemed a prescription of smoking cessation medication in the period 2009–2015. If we take the estimated smoking prevalence into account, only 36–55% of the expected active smokers with a COPD–diagnosis collected smoking cessation medication. Female sex and being employed were both associated with increased likelihood of collecting smoking cessation medication.

Making smoking cessation treatment equally available to all individuals requires more than offering free advice on smoking cessation. Socioeconomic factors must be considered before the type of smoking cessation treatment is chosen. Individually tailored smoking cessation treatment should be offered to patients with COPD to reduce the social inequality observed in this group. One way could be to permanently include smoking cessation medication in the national reimbursement system to lower the prices. To increase the success rates of smoking cessation, medical treatment should be combined with professional counseling.

Smoking cessation is the cornerstone in the treatment of COPD, especially in younger patients since their lungs seem particularly vulnerable to the damaging effects of tobacco use. Health promotion is a core task in general practice. Helping people to quit smoking is an action with considerable impact on public health. Our study emphasizes that there is room for better support and motivation to help young people with COPD to successful smoking cessation.

## Supplementary information

REPORTING SUMMARY

## Data Availability

The data used in the study are available from the corresponding author upon e-mail request.
